# S12-3 School-based physical activity promotion in a cross-cultural context: interventions from France and Spain

**DOI:** 10.1093/eurpub/ckad133.060

**Published:** 2023-09-11

**Authors:** Hisham Bachouri-Muniesa, Nicolas Fabre, Alberto Aibar, Lena Lhuisset, Sonia Asún-Dieste, Julien Bois, José A Julián-Clemente, Eduardo Ibor, Javier Zaragoza

**Affiliations:** University of Zaragoza; University of Pau and Pays de l’Adour; University of Pau and Pays de l’Adour; University of Zaragoza; University of Pau and Pays de l’Adour; University of Zaragoza; University of Pau and Pays de l’Adour; University of Zaragoza; University of Zaragoza; University of Zaragoza

## Abstract

**Purpose:**

School-based interventions seems to be effective to promote active lifestyles among youth but recent literature shows that these interventions have a lack of effectiveness and very few report data on its sustainability. In the same way, few of these interventions are being replicated in different contexts than their beginning. Therefore, the balance between the adaptability and fidelity of a programme in a new context is nowadays an implementation gap.

**Project:**

For this reason, the 2PASS-4Health (Promoting PA in Secondary Schools for Health) project has created a collaborative network between different European universities with the purpose of promoting Health-Enhancing Physical Activity (HEPA) in adolescents in a school-based environment. This project was made to identify the good practices and the main problems linked to the design, implementation, evaluation, and sustainability of interventions aimed at HEPA promotion. In this line, two school-based (effective) interventions in a cross-cultural context have been disseminated and developed, at the same time, both in France and Spain respectively to examine the key parts in the process of fidelity, adaptability and sustainability of them.

The intervention developed in France, took place in Tarbes, a little city in the south of the country. In the other side of the Pyrenees, the other intervention took place in Jaca (Spain), in the north of Aragon’s region. Both interventions adopted a multi-level approach involving all parts of the educational community: students, families, teachers and policymakers. The research team (all together) with the school’s staff developed the interventions by a co-creational process helped by local policymakers. As a result of these interventions, an intervention guide was developed. This tool can be used by stakeholders to disseminate and adapt these school-based interventions to make their effects sustainable over time (sustainability).

**Conclusion:**

The collaborative line of this project renders visible the possibility of connecting cross-cultural educational contexts, through common elements in similar school-based interventions, with the aim of creating environments that promote HEPA.

